# HAS2-Ezrin-ER axis plays a role in acquired antiestrogen resistance of ER-positive breast cancer

**DOI:** 10.3389/fphar.2022.1031487

**Published:** 2022-10-31

**Authors:** Xiaodan Sun, Fen Tang, Qian Guo, Yiwen Liu, Yiqing He, Yan Du, Feng Gao, Guoliang Zhang, Cuixia Yang

**Affiliations:** ^1^ Department of Clinical Laboratory, Shanghai Sixth People’s Hospital Affiliated to Shanghai Jiao Tong University School of Medicine, Shanghai, China; ^2^ Department of Molecular Biology, Shanghai Sixth People’s Hospital Affiliated to Shanghai Jiao Tong University School of Medicine, Shanghai, China; ^3^ Department of Breast Surgery, Shanghai Sixth People’s Hospital Affiliated to Shanghai Jiao Tong University School of Medicine, Shanghai, China

**Keywords:** endocrine resistance, HAS2, Ezrin, ERα, antiestrogens

## Abstract

The development of endocrine resistance is a major clinical problem in estrogen receptor-positive (ER^+^) breast cancer (BrCa) treatment, in which how cancer cells acquire resistance remains obscure. Hyaluronan synthase 2 (HAS2) is the most critical synthase in producing hyaluronan and is well known for its involvement in cancer growth, metabolism and metastasis. Recent evidence has proved that HAS2 is involved in cellular acquired resistance to drug therapy in BrCa. In this work, we first observed that HAS2 expression was decreased in the endocrine-resistant ER^+^ BrCa cells. Further knocking-out experiments confirmed that the loss of HAS2 in parental ER^+^ BrCa cells resulted in a following antiestrogen resistance. Next, we found that the HAS2-loss could induce an upregulation of Ezrin, a member of the membrane cytoskeletal protein family who plays key roles in cellular signal transduction. Notably, we identified that the increase of Ezrin induced by HAS2-loss could inhibit the ERα expression and augment antiestrogen resistance, suggesting that a HAS2-Ezrin-ER axis may be associated with the acquirement of endocrine resistance in ER^+^ BrCa cells. Finally, knockdown or inhibition of Ezrin could restore the sensitivity of endocrine-resistant cells to antiestrogens treatment by activating ERα signaling. Taken together, our findings unraveled a novel HAS2-Ezrin-ER route in regulating the sensitivity of ER^+^ BrCa cells to antiestrogens, in which Ezrin may be a potential target in endocrine therapy.

## Introduction

Acquired resistance to endocrine therapy is a major challenge for estrogen receptor-positive (ER^+^) breast cancer (BrCa) and leads to unfavorable clinical outcome (Hanker et al., 2020). Clinical retrospective studies have proved that the ER expression was reduced at relapse sites after long-term antiestrogens treatment (Johnston et al., 1995; Hart et al., 2015). Until now, the mechanism underlying endocrine resistance due to the reduction of ER expression is poorly understood. Hyaluronan synthase 2 (HAS2) is a key hyaluronan (HA) synthase embedded in cell membrane (Watanabe and Yamaguchi, 1996; Camenisch et al., 2000). Besides synthesizing HA, HAS2 is also an important modulator of tissue microenvironment involved in restoring tissue homeostasis (Rauhala et al., 2018). The expression of HAS2 is sensitive to survival signals in cell microenvironment, such as glucocorticoid (Zhang et al., 2000; Saavalainen et al., 2005; Pasonen-Seppänen et al., 2008), prostaglandins (Sussmann et al., 2004), and cytokines (Tammi et al., 2011). Most importantly, mountains of evidence have demonstrated that HAS2 is closely associated with various types of cancer progression. For example, studies have showed that HAS2 mRNA is abundantly expressed in the hormone-negative breast cancer cell lines (Heldin et al., 2014). In addition, evidences also proved that HAS2 is involved in BrCa cells phenotype plasticity and chemotherapy drug-resistance (Yang et al., 2013; Chanmee et al., 2014). Our recent report also identified that HAS2 is involved in cellular acquired resistance to PI3Kα-inhibitory chemotherapy in BrCa (Yang et al., 2020). It seems likely that the role of HAS2 in development of endocrine resistance should be noted. Unfortunately, to our knowledge, few studies have been reported in such area.

It is well known that HAS2 is a membrane-bound protein with distinct intracellular and extracellular functions (Passi et al., 2019). The extracellular segment is responsible for HA synthesis while the intracellular one is related to the functions of cytoskeleton proteins (Vigetti and Passi, 2014). For example, intracellular cytoskeletal proteins Ezrin-Radixin-Moesin (ERM) are required for cell survival and movement that include cytoskeleton reorganization and cell migration. We recently reported that Ezrin existed in the interconnected network of HAS2 mediated resistance to PI3Kα inhibitors with enhanced ER signaling (Yang et al., 2020), suggesting its functional role in regulating ER activity. As it is widely accepted that the decrease of ER is an indicator of antiestrogens resistance, we herein hypothesized that HAS2-Ezrin-ER axis may be involved in the acquired antiestrogen resistance of ER^+^ BrCa. In addition, previous studies have implicated that the AKT/mTOR signaling pathway and the MAPK pathway are potential modulators of endocrine resistance in BrCas (Zhang et al., 2011; Araki and Miyoshi, 2018). We therefore sought to explore whether the HAS2-Ezrin-ER axis regulates these pathways. Besides, transcription factor FOS and PBX1 have been reported to be indicators of the activation of ER (Cheung et al., 2005; Toska et al., 2017), we then carried out studies to determine the function of these transcription factors and mechanism of HAS2 in triggering resistance in cancer cells through the regulation of ER-dependent transcriptional response.

In this study, we aimed to investigate the role of HAS2 in ER^+^ BrCa cells under long-term exposure to antiestrogens treatment. We first observed that HAS2 expression was decreased in the endocrine-resistant cells and knockout of HAS2 in parental ER^+^ BrCa cells could reduce the sensitivity to antiestrogens. Next, we demonstrated that Ezrin was increased in the resistant cells and could be negatively regulated by HAS2. Finally, inhibition of Ezrin in resistant cells could rescue the ER activity, leading to the restoration of antiestrogens sensitivity. Our results suggested that a HAS2-Ezrin-ER axis may be engaged in the endocrine resistance of ER^+^ BrCa and Ezrin could be a potential therapeutic target.

## Materials and methods

### Cell culture

The human breast cancer cell lines MCF7 and T47D were purchased from the Cell Bank of Chinese Academy of Sciences (Shanghai, China). MCF7 cells were cultured in MEM medium (Gibco, Thermo Fisher Scientific, United States), supplemented with 10% fetal calf serum, 1× non-essential amino acid (NEAA), 0.01 mg/ml bovine insulin, 100 U/ml penicillin and 100 mg/ml streptomycin. T47D cells were cultured in RPMI-1640 medium, supplemented with 10% fetal calf serum, 0.0075 mg/ml bovine insulin, 100 U/ml penicillin and 100 mg/ml streptomycin. All cells were cultured in a humidified incubator with 5% CO_2_ at 37°C and grown to 85% confluence for the experiments. All the cell lines were tested negative for *mycoplasma*.

### Generation of resistant cell lines

MCF7 and T47D cells were maintained in the phenol-red-free media supplemented with 5% charcoal/dextran-treated FBS. The resistant cell lines were generated by long-term treatment with 1 μM tamoxifen or fulvestrant (MedChem Express, United States) as previously reported (Thrane et al., 2015; Zhu et al., 2018).

### Hyaluronan synthase 2 knockout and overexpression

HAS2 in MCF7 cells was knocked out using CRISPR/Cas9. Plasmids targeting HAS2 were purchased from Santa Cruz (sc-401032, United States). The guide RNA vector was cloned into a pCas-Guide vector which expresses Cas9 behind CBh and U6 promoters. To target the HAS2, a guide RNA vector including the target sequences (CTC​GCA​ACA​CGT​AAC​GCA​AT, TCC​AGT​GAT​AAT​CGC​TTC​GT, and ACCTACG AAGCGATTATCAC) was prepared following the depositor’s instruction. This vector was co-transfected with the donor template including homologous arms and a functional GFP-puromycin cassette using UltraCruz^®^ Transfection Reagent (sc-395739) as the delivery reagent. MCF7 cells were passaged at 48 h post-transfection for three passages. Cells were treated with 2 μg/ml puromycin until the pooled population of all puromycin-resistant cells was expanded. Then, the CAS9/Control cells and HAS2 KO cells were used for experiments.

HAS2 was overexpressed in MCF7 TamR/FulR cells by lentiviral transduction as previously described (Zhang et al., 2016). The pCMVIE-IRES-HAS2 vector encoding HAS2 was purchased from Hanbio (Shanghai, China). Stable HAS2-expressing cells were selected using puromycin.

### Cell proliferation

The proliferation of BrCa cells was determined by the CCK-8 assay (Dojindo, Japan). Cells were seeded into 96-well plates (5 × 10^3^ cells/well) and treated with 1 μM tamoxifen, 1 μM fulvestrant, or 0.5 μM Ezrin-inhibitor (NSC668394, MedChem Express, United States) for the indicated time. Then, the CCK-8 reagent (20 μl) was added to each well and incubated at 37°C for 2 h. The optical density at 490 nm was measured using an automatic microplate reader (BioTek Epoch, United States).

### 5-Ethynyl-2′-deoxyuridine assay

Cell proliferation was also analyzed with a Cell-Light EdU DNA Cell Proliferation kit (Beyotime Institute of Biotechnology, China). Naive and endocrine-resistant BrCa cells were seeded in 96-well plates (1 × 10^4^ cells/well) and treated with tamoxifen. After incubation for 48 h, 10 mM EdU was added and incubated at 37°C for 2 h. Cells were fixed with 4% paraformaldehyde and stained with azide 594 (30 min, for proliferating cells) and Hoechst 33,342 (10 min, for nuclei) at room temperature. Images were captured by a fluorescence microscope (Nikon, Japan). The percentage of proliferating cells was calculated using ImageJ software (National Institutes of Health, United States).

### Western blot

Total cell lysates were collected, and equal quantities of protein were separated *via* 10% sodium dodecyl sulfate-polyacrylamide gel electrophoresis (SDS-PAGE) and blotted onto a polyvinylidene difluoride (PVDF) membrane. PVDF membranes were blocked with Tris-buffered saline with tween 20 (TBST) containing 5% skimmed milk powder for 1 h, and incubated with primary antibodies against HAS2 (Abcam, ab131364), pAKT308 (Cell Signaling Technology, 13038T), pAKT473 (Cell Signaling Technology, 4060T), pERK (Cell Signaling Technology, 4370P), ERK (Cell Signaling Technology, 4691P), Ezrin (Cell Signaling Technology, 3145s), ERα (Abcam, ab32063), or pERα (Cell Signaling Technology, 2511S) at 4°C overnight. Then, the membranes were washed with TBST buffer three times (10 min each time) and incubated with horseradish peroxidase (HRP)-conjugated polyclonal secondary antibody for 1 h. Finally, the membranes were developed using the enhanced plus chemiluminescence assay (Thermo Fisher Scientific, United States) according to the manufacturer’s instructions.

### Quantitative real-time PCR

Total RNA was extracted from cultured cells using RNAiso Plus (Takara, Japan). The RNA concentration was measured using the NanoDrop system (Thermo Fisher Scientific, United States). Then, total RNA (1 µg) was reverse-transcribed using the PrimeScript™ RT Reagent kit with gDNA Eraser (Takara, Japan). qPCR assays were performed with SYBR Green mix (Takara, Japan) according to the manufacturer’s protocol. The relative mRNA expression was analyzed by the change-in-threshold (2^−ΔΔCT^) method of the specific gene over the housekeeping gene GAPDH. Sequences of primers used were shown in the Supplementary Table S1.

### Human breast cancer samples

This study was approved by the ethics committee of Shanghai Jiao Tong University Affiliated Sixth People’s Hospital. Written informed consent was obtained from all individual participants in accordance with the Declaration of Helsinki of the World Medical Association. We analyzed tissue specimens (*n* = 18) obtained from patients diagnosed with BrCa in Shanghai Jiao Tong University Affiliated Sixth People’s Hospital between 1 November 2020, and 31 July 2021. TNM staging system was used to stage tumors.

### Immunohistochemistry

Tissue samples from BrCa patients were fixed in 10% buffered neutral formalin, embedded in paraffin, and mounted on glass slides. After dewaxing and dehydration, antigens were recovered and endogenous peroxidase activity and nonspecific binding were inhibited. Then, primary antibodies against HAS2 (Abcam), ERα (Abcam) or Ezrin (Cell Signaling Technology) were added, and the slides were incubated at 4°C overnight. After washing with PBS, the slides were incubated with biotinylated secondary antibodies for 1 h, followed by streptavidin-ABC at room temperature. Then, the slides were developed with a 2,4-diaminobutyric acid (DAB) Substrate Kit and counterstained with hematoxylin. The intensities of HAS2, ERα, and Ezrin were quantitatively analyzed using Image-Pro Plus 6.0 software (Media Cybernetics, United States).

### Immunofluorescence

The expression of Ezrin in cells was also determined by an immunofluorescence assay. Cells were fixed in 4% paraformaldehyde, permeabilized with Triton X-100, and blocked in 5% BSA. Then, cells were incubated with primary antibodies against ERα or Ezrin overnight at 4°C in incubation fluid (Dako, Denmark). On the second day, cells were washed with PBST three times (10 min each time) and then incubated with the secondary antibody conjugated with Alexa Fluor 647 (Abcam, ab150083) for 1 h at room temperature. Then, cells were stained for F-actin through the incubation with Phalloidin-iFluor 488 reagent (Abcam, ab176573) for 90 min at room temperature. Cells were washed in PBST three times (10 min each time). Images were analyzed using a confocal microscopy (Nikon A1, Japan).

### Knockdown of Ezrin using small-interfering RNAs

The expression of Ezrin was downregulated by siRNAs. SiRNA transfection was performed using a riboFECT^TM^CP (RiboBio, Shanghai, China) kit according to the manufacturer’s protocol. 100 nM siRNA targeting Ezrin was used (pool of three sequences: 5ʹ-GGA​TTA​CTG​CGT​CGA​TTC​A-3ʹ, 5ʹ-CCA​TGG​CTT​TCC​CGG​ATA​A-3ʹ, and 5ʹ-GTCGAG GCATGGAGTTCAA-3ʹ).

### Statistical analysis

Statistical analysis was performed by GraphPad Prism 6 (GraphPad Software, United States). The significance of differences among groups was determined by *t*-test or Mann-Whitney test accordingly. Statistically significance was considered as *p* < 0.05.

## Results

### Decrease of hyaluronan synthase 2 was associated with antiestrogen resistance in ER^+^ breast cancer

**FIGURE 1:**
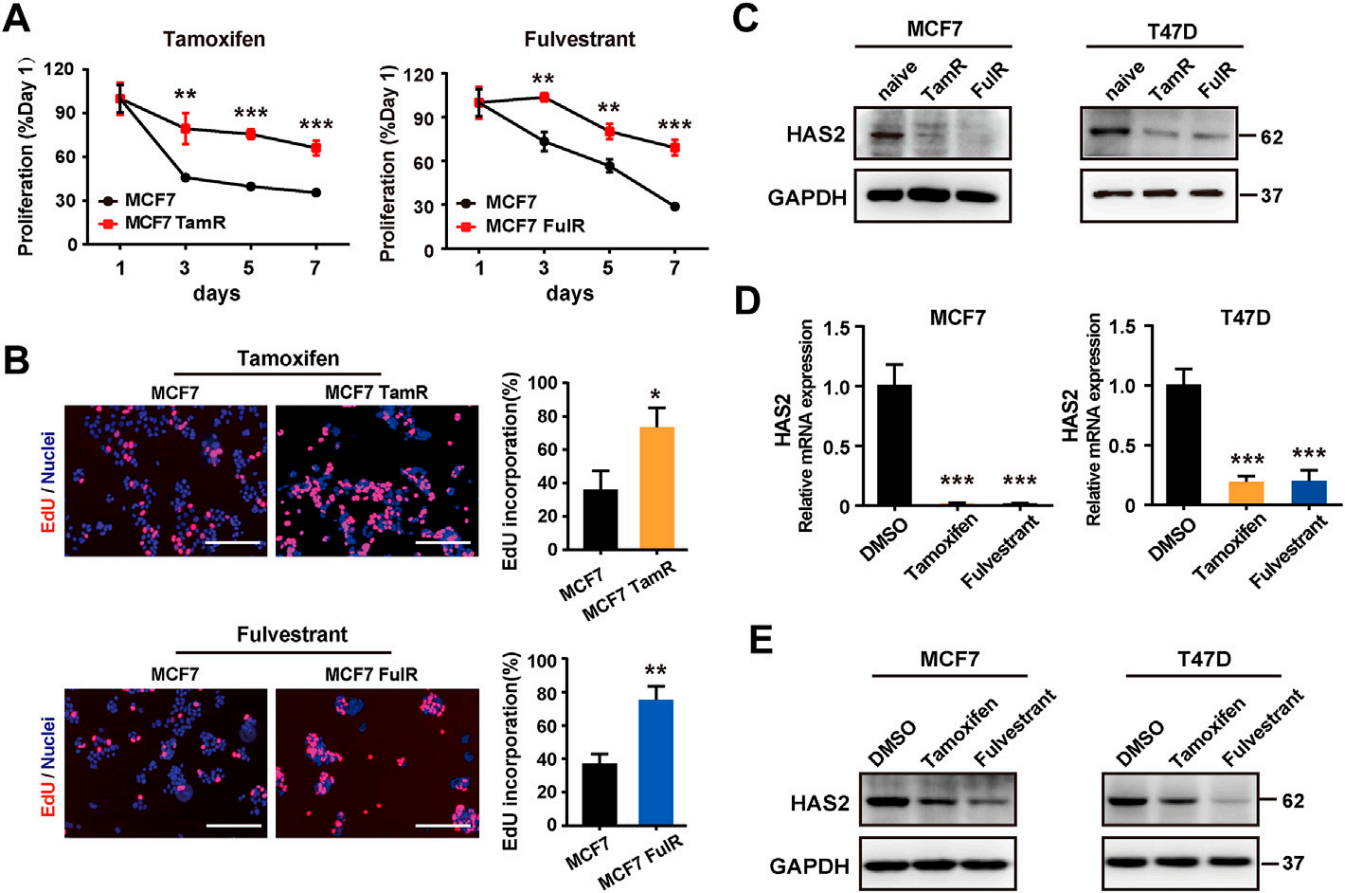
Decrease of HAS2 was associated with antiestrogen resistance in ER^+^ breast cancer. **(A)** Proliferation of endocrine-resistant MCF7 cells was compared with their sensitive counterparts upon antiestrogens treatment (1 μM) at 1, 3, 5, and 7 days. **(B)** An EdU (5-ethynyl-2′-deoxyuridine) assay was performed to assess DNA synthesis. EdU (red) and Hochest 33,342 (blue) were used to stain proliferative cells and nuclei, respectively. Representative images of at least three experiments were shown. Scale bar, 200 µm. Quantitation of the EdU incorporation (%) was shown. **(C)** The levels of HAS2 in resistant BrCa cells (MCF7 and T47D) and their parental counterparts were determined by western blot. **(D)** The mRNA levels of HAS2 in naive MCF7 and T47D cells upon DMSO, tamoxifen (1 μM), or fulvestrant (1 μM) treatment for 3 days were detected by RT-qPCR. **(E)** Immunoblotting analysis of HAS2 expression in naïve MCF7 and T47D cells treated with DMSO, tamoxifen (1 μM), or fulvestrant (1 μM) for 3 days. Statistical analysis: *t*-test. Values were presented as the mean ± SD. *p < 0.05, **p < 0.01, ***p < 0.001.

Although HAS2 expression is sensitive to estrogen (Sugiura et al., 2010; Akgul et al., 2012), its contribution to antiestrogen resistance is poorly understood. To determine whether HAS2 is associated with antiestrogen resistance, we first established tamoxifen- or fulvestrant-resistant ER^+^ BrCa cell lines (referred to as TamR and FulR, respectively) as previously reported (Thrane et al., 2015; Zhu et al., 2018). As expected, the results showed that the proliferation of MCF7 TamR and MCF7 FulR was higher than native cells upon antiestrogen treatment (Figures 1A,B; Supplementary Figure S1). Notably, a significant decrease of HAS2 was found in endocrine-resistant MCF7 and T47D BrCa cells compared with their endocrine-sensitive parental cells (Figure 1C). We next observed the effects of antiestrogens treatment on HAS2 expression in native ER^+^ BrCa cells. Similarly, the results showed that both the mRNA and protein expression levels of HAS2 were significantly reduced upon short-term tamoxifen or fulvestrant treatment (Figures 1D,E; Supplementary Figure S2). Collectively, these results suggested that there was a correlation between the decrease of HAS2 and the development of endocrine resistance.

### Hyaluronan synthase 2 regulated the sensitivity of estrogen receptor-positive breast cancer cells to antiestrogens

**FIGURE 2:**
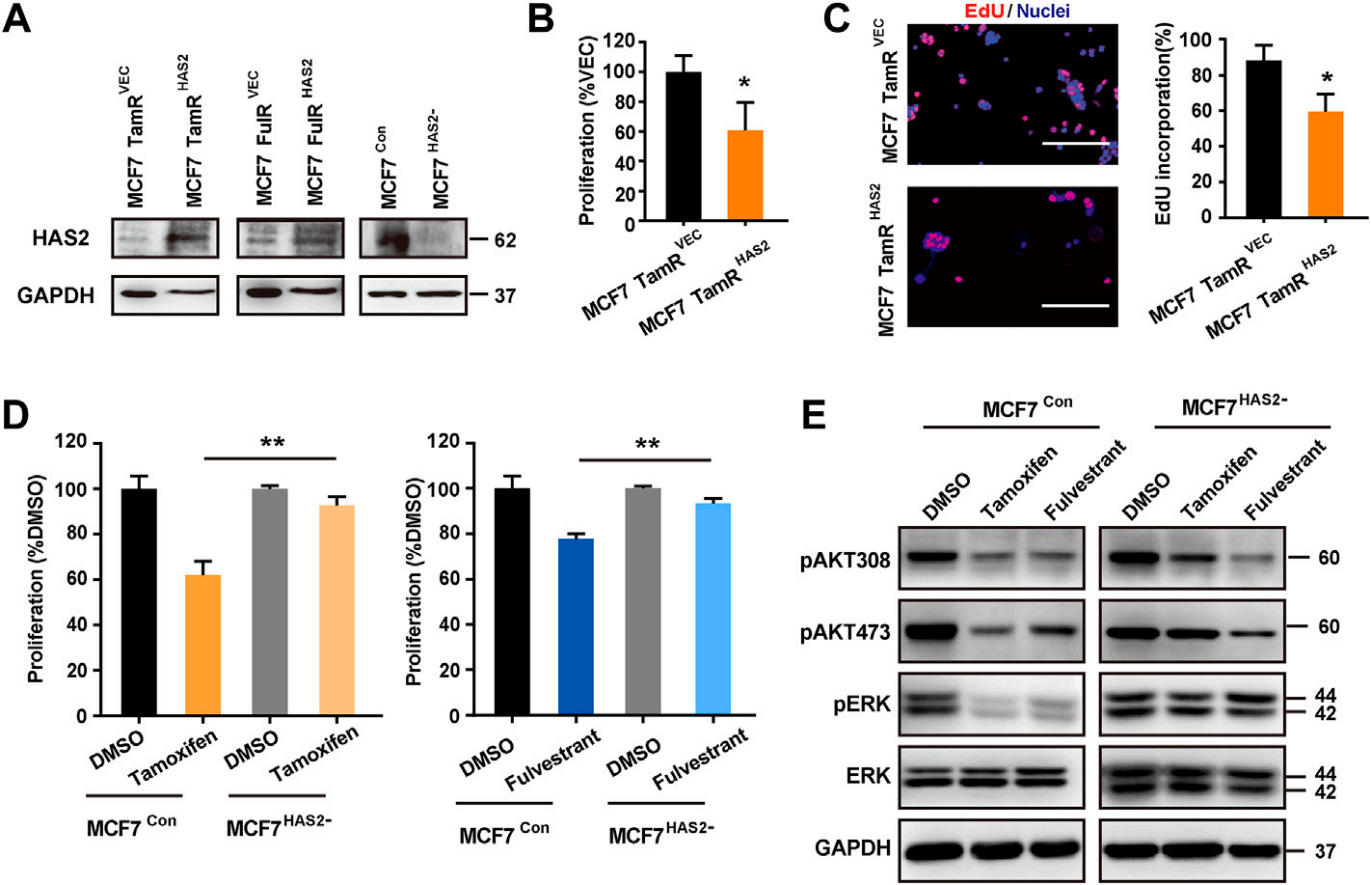
HAS2 regulated the sensitivity of ER^+^ breast cancer cells to antiestrogens. **(A)** Immunoblotting analysis of HAS2 expression in endocrine-resistant MCF7 cells (MCF7 TamR and MCF7 FulR) with HAS2 overexpression and naïve MCF7 cells with HAS2 knockout. **(B)** The proliferation of MCF7 TamR cells with (MCF7 TamR^HAS2^) or without (MCF7 TamR^Vec^) HAS2 overexpression was determined by CCK8 assay after being treated with tamoxifen (1 μM) for 3 days. **(C)** An EdU assay was performed to assess DNA synthesis. Representative images of at least three experiments were shown. Scale bar, 200 µm. Quantitation of the EdU incorporation (%) was shown. **(D)** The effect of HAS2 knockout on the sensitivity of MCF7 cells to tamoxifen or fulvestrant was determined by a CCK8 kit. MCF7^Con^ and MCF7^HAS2-^ cells were treated with tamoxifen or fulvestrant (1 μM) for 3 days. **(E)** The effects of HAS2 loss on Akt and MAPK signaling pathways upon tamoxifen or fulvestrant treatment were determined by western blot. Statistical analysis: *t*-test. Values were presented as the mean ± SD. *p < 0.05.

As HAS2 expression was decreased in TamR/FulR cells compared with the parental cells, we further investigated the effects of HAS2 on the sensitivity to antiestrogens therapy. We first constructed the stable HAS2-overexpressed endocrine-resistant cells (MCF7 TamR^HAS2^/MCF7 FulR^HAS2^) and HAS2-knockout parental cells (MCF7^HAS2-^), respectively (Figure 2A). As shown in Figures 2B,C, the overexpression of HAS2 significantly promoted the sensitivity of MCF7 TamR cells to tamoxifen, while the knockout of HAS2 in MCF7 cells inhibited the susceptibility to tamoxifen or fulvestrant (Figure 2D). Given that AKT and MAPK/ERK signaling pathways are involved in cell survival and proliferation (Mishra et al., 2010), we next observed the effect of HAS2 on the activation of AKT and ERK. The results showed that tamoxifen or fulvestrant could significantly inhibit the phosphorylation of AKT and ERK in MCF7 cells, whereas HAS2 knockout attenuated the decreased effect of tamoxifen or fulvestrant (Figure 2E). Together, these results suggested that the loss of HAS2 could reduce the sensitivity of ER^+^ BrCa cells to antiestrogens.

### Hyaluronan synthase 2 decrease upregulated ezrin expression in response to antiestrogens in estrogen receptor-positive breast cancer cells

**FIGURE 3:**
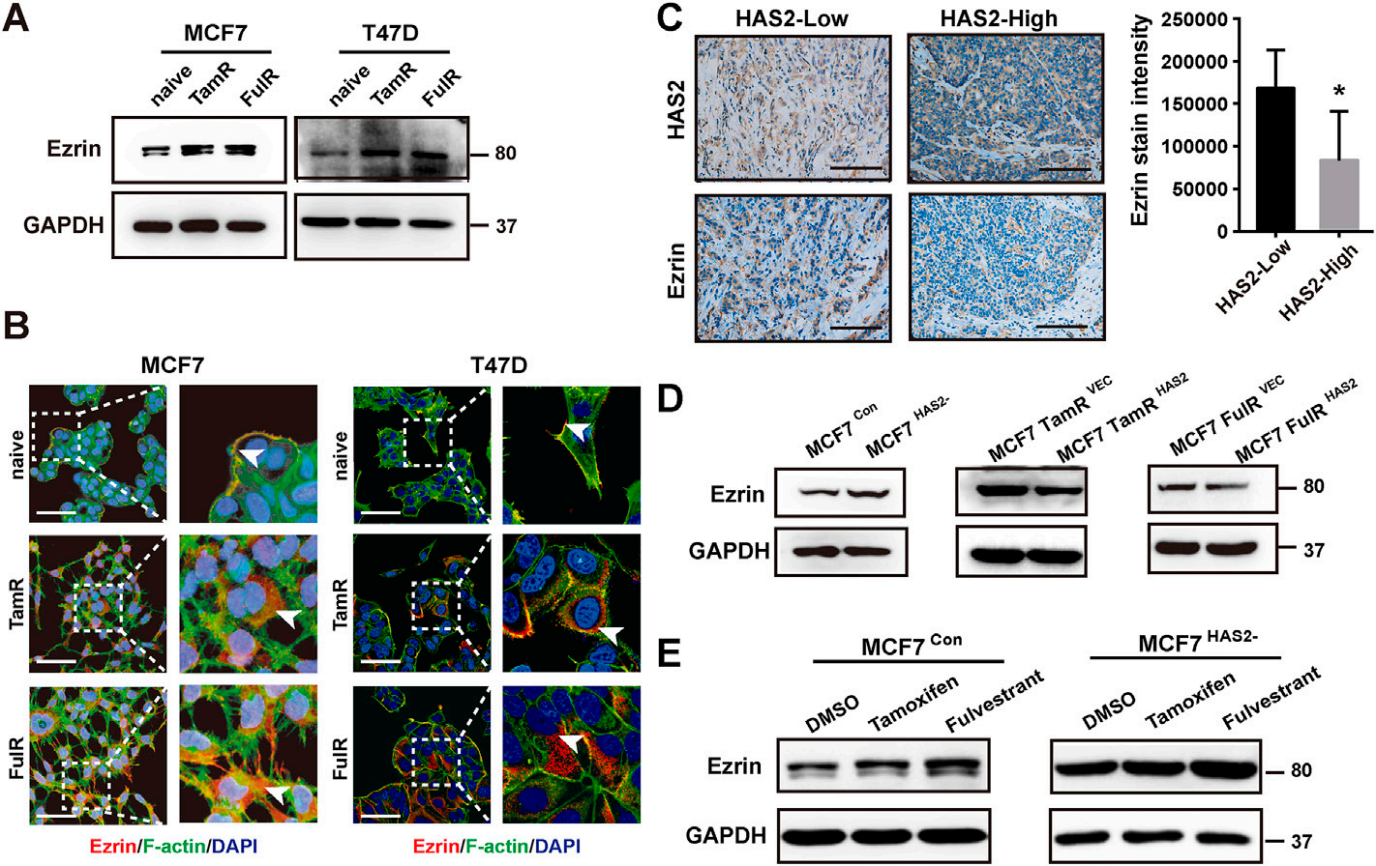
HAS2 decrease upregulated Ezrin expression in response to antiestrogens in ER^+^ breast cancer cells. **(A)** Immunoblotting analysis of Ezrin expression was performed in sensitive and resistant cells as indicated. **(B)** The levels of Ezrin in endocrine-resistant MCF7 and T47D cells were observed by immunofluorescence. Cells were stained with Ezrin antibody (red), Phalloidin-iFluor 488 reagent for F-actin (green), and DAPI for nuclei (blue). Scale bars, 50 µm. **(C)** The levels of Ezrin and HAS2 in ER^+^ human breast cancer tissues were evaluated by immunohistochemistry. Significant (*p < 0.05, Mann-Whitney test) decrease of Ezrin staining was observed in HAS2^high^ breast cancer patients (*n* = 7) compared with HAS2^low^ patients (*n* = 5). **(D)** The changes of Ezrin levels in naïve MCF7 cells with HAS2 knockout and endocrine-resistant MCF7 cells with HAS2 overexpression. **(E)** MCF7 cells with or without HAS2 knockout were stimulated by tamoxifen or fulvestrant (1 μM) for 3 days. Then the expression of Ezrin was analyzed by western blot.

We recently reported that the cytoskeletal protein Ezrin may be a downstream effector in the network of HAS2 induced resistance to PI3Kα inhibitors (Yang et al., 2020) and could be regulated in response to estrogen (Stemmer-Rachamimov et al., 2001; Song et al., 2005). We next investigated the role of Ezrin in the acquisition of resistance to antiestrogens in ER^+^ BrCa cells. First, we found that the Ezrin expression was increased in resistant cells (Figures 3A,B). To identify the association between HAS2 and Ezrin, we then detected the Ezrin levels in BrCa patients with low or high level of HAS2 by immunohistochemistry. As shown in Figure 3C, Ezrin expression was high in BrCa tissues with low-level of HAS2, suggesting a negative correlation between HAS2 and Ezrin in BrCa patients. Next, to determine whether HAS2 could regulate Ezrin, we detected the effect of overexpression or knockout of HAS2 on Ezrin expression. Our results revealed that knockout of HAS2 in MCF7 parental cells increased Ezrin expression, while the overexpression of HAS2 downregulated Ezrin expression in MCF7 TamR or FulR cells (Figure 3D). Further, the role of HAS2 in regulating the expression of Ezrin upon antiestrogen treatment was studied. The results showed that the knockout of HAS2 weakened the increase of Ezrin expression, which was induced by antiestrogen treatment (Figure 3E). These data implied that HAS2 could negatively regulate Ezrin expression in ER^+^ breast cancer.

### Hyaluronan synthase 2-Ezrin-ER axis regulated antiestrogen sensitivity

**FIGURE 4:**
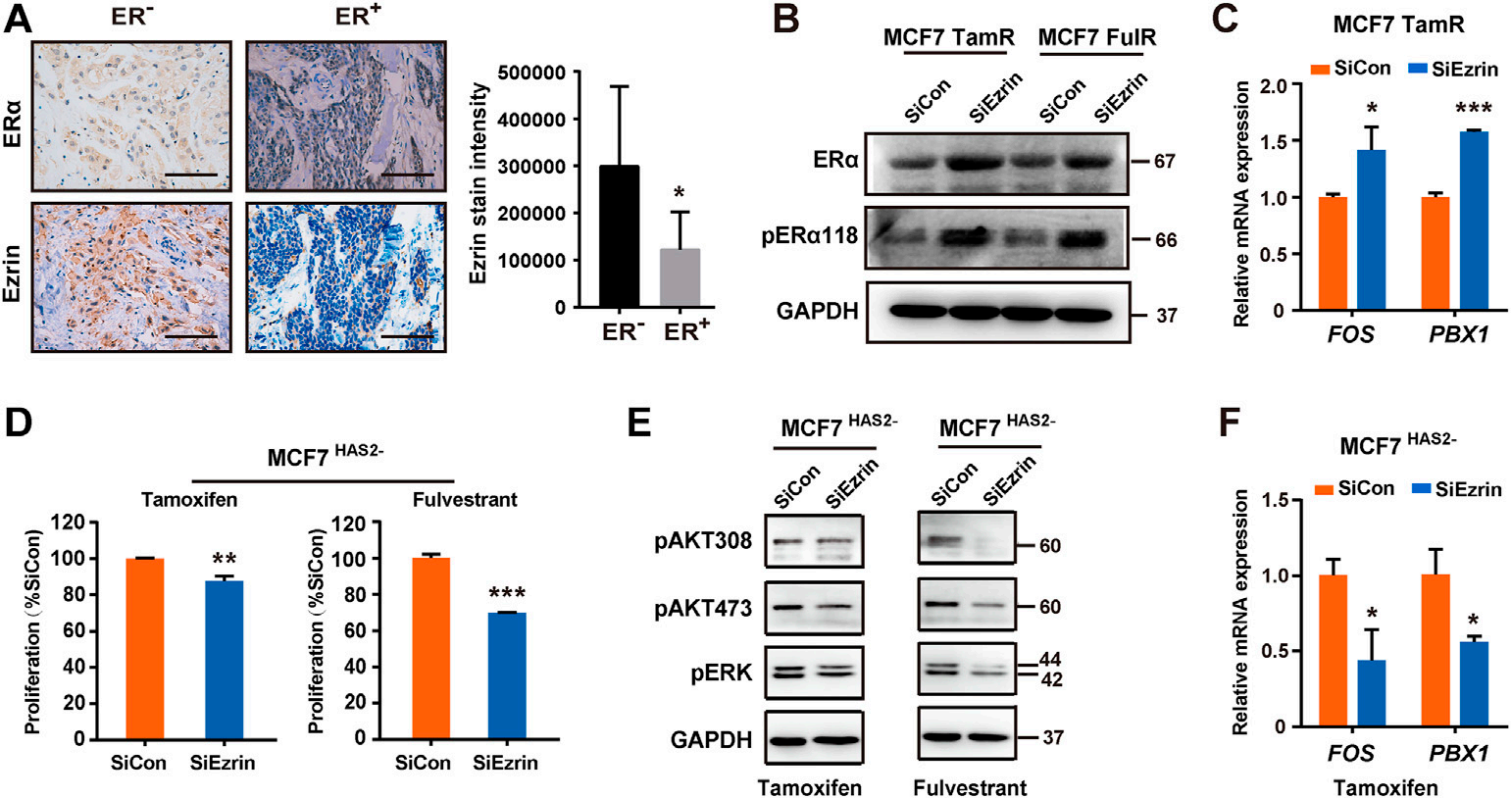
HAS2-Ezrin-ER axis regulated antiestrogen sensitivity. **(A)** The levels of ER and Ezrin in human breast cancer tissues were evaluated by immunohistochemistry. Significant (*p < 0.05, Mann-Whitney test) decrease of Ezrin staining was observed in patients with ER^+^ breast cancer (*n* = 12) compared with ER^−^ patients (*n* = 6). **(B)** Knockdown of Ezrin promoted ER expression and activation in resistant MCF7 cells. **(C)** The effects of Ezrin knockdown on the mRNA levels of FOS and PBX1 in MCF7 TamR cells were determined by real-time PCR. **(D)** MCF7 ^HAS2-^ cells were transfected with siEzrin or control siRNAs, and exposed to 1 μM of tamoxifen or fulvestrant for 3 days. Then, the proliferation of cells was detected using a CCK-8 assay. **(E)** The AKT and MAPK signaling pathways were analyzed in MCF7 ^HAS2-^ cells transfected with siEzrin or control siRNAs in the presence of 1 μM antiestrogens for 3 days. **(F)** MCF7^HAS2-^ cells were transfected with siEzrin or control siRNAs, and treated with tamoxifen or fulvestrant (1 μM) for 3 days. Then, the mRNA levels of FOX and PBX1 were evaluated. Statistical analysis: *t*-test. Values were presented as the mean ± SD. *p < 0.05, **p < 0.01, ***p < 0.001.

It is well known that the reduction of ERα is an indicator of endocrine resistance (Johnston et al., 1995). As Ezrin was increased in resistant ER^+^ BrCa cells as mentioned above, we next investigated the relation between the Ezrin and ERα in BrCa by immunohistochemistry. Our results showed that the Ezrin expression in ER^−^ BrCa was higher than that in ER^+^ BrCa (Figure 4A), suggesting that there may be a negative correlation between Ezrin and ERα in BrCa patients. We then tried to explore whether Ezrin could regulate the ER expression and activation. Data indicated that the down-regulation of Ezrin in MCF7 TamR or FulR cells increased the expressions of ERα and phosphorylated ERα (Figure 4B). Given that FOS and PBX1 are known indicators of the activation of ER signaling (Fallah et al., 2017; Toska et al., 2017), we next assessed the effect of Ezrin on the transcription of these ER-dependent target genes. As expected, the knockdown of Ezrin could increase the mRNA levels of FOS and PBX1 in endocrine-resistant cells (Figure 4C), suggesting that Ezrin could regulate ER expression and activation. We next determined whether the Ezrin could regulate the acquisition of antiestrogens resistance. As shown in Figures 4D,E, the increase of resistance to antiestrogens in MCF7^HAS2-^ cells was significantly suppressed by Ezrin knocking down, indicating the contribution of Ezrin to antiestrogens resistance induced by HAS2 decrease. Consistently, down-regulation of Ezrin suppressed the ER activation indicators (PBX1, FOS) in MCF7^HAS2-^ cells upon tamoxifen treatment (Figure 4F). Taken together, these data suggested that there might be a HAS2-Ezrin-ER axis in regulating the acquired antiestrogen resistance of ER^+^ breast cancer.

### Inhibition of Ezrin restored the sensitivity of estrogen receptor-positive breast cancer cells to antiestrogens

**FIGURE 5:**
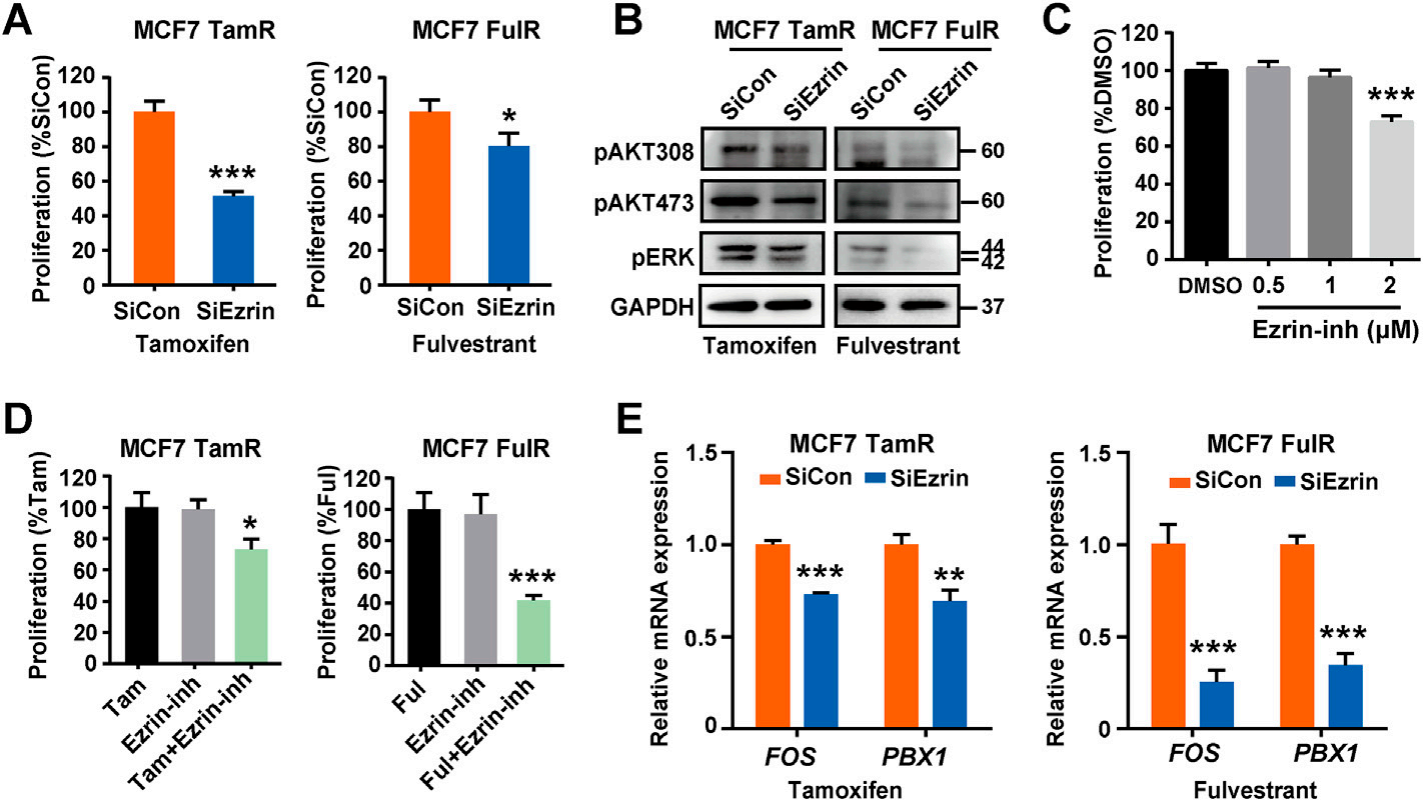
Inhibition of Ezrin restored the sensitivity of ER^+^ BrCa cells to antiestrogens. **(A)** The changes of cell sensitivity to tamoxifen or fulvestrant in resistant MCF7 cells with Ezrin knockdown were determined by a CCK-8 assay. **(B)** Resistant MCF7 cells were transfected with siEzrin or control siRNAs. Then, the changes of cell survival signaling in the presence of tamoxifen or fulvestrant were determined by immunoblotting. **(C)** The effect of Ezrin inhibitor (Ezrin-inh) at different concentrations on the proliferation of naïve MCF7 cells. **(D)** The influence of Ezrin inhibitor on the sensitivity of resistant MCF7 cells to tamoxifen or fulvestrant was determined using a CCK-8 assay. **(E)** Resistant MCF7 cells were transfected with siEzrin or control siRNAs, and treated with tamoxifen or fulvestrant (1 μM) for 3 days. Then, the mRNA levels of FOX and PBX1 were evaluated. Statistical analysis: *t*-test. Values were presented as the mean ± SD. *p < 0.05, **p < 0.01, ***p < 0.001.

As Ezrin played a key role in HAS2 induced endocrine resistance, we next examined the effect of Ezrin knockdown on the proliferation of resistant cells. The results showed that the knockdown of Ezrin by siRNAs could reduce the cell survival of MCF7 TamR or FulR cells upon antiestrogens treatment (Figures 5A,B). To further verify the inhibiting effect of Ezrin, an Ezrin inhibitor that blocks Ezrin phosphorylation was used. We preliminarily observed the cytotoxicity of the Ezrin inhibitor. As shown in Figure 5C, the Ezrin inhibitor had no obvious cytotoxic effect at both concentrations of 0.5 μM and 1 μM, in which 0.5 μM was selected in the following experiments. The results revealed that the Ezrin inhibitor could increase the sensitivity of endocrine-resistant cells to tamoxifen or fulvestrant (Figure 5D). Similarly, the ER-dependent transcription genes FOS and PBX1 were accordingly reduced upon tamoxifen or fulvestrant stimulation after Ezrin knockdown (Figure 5E). Collectively, these results showed that suppressing Ezrin could restore the sensitivity of endocrine-resistant cells to antiestrogens.

## Discussion

**FIGURE 6:**
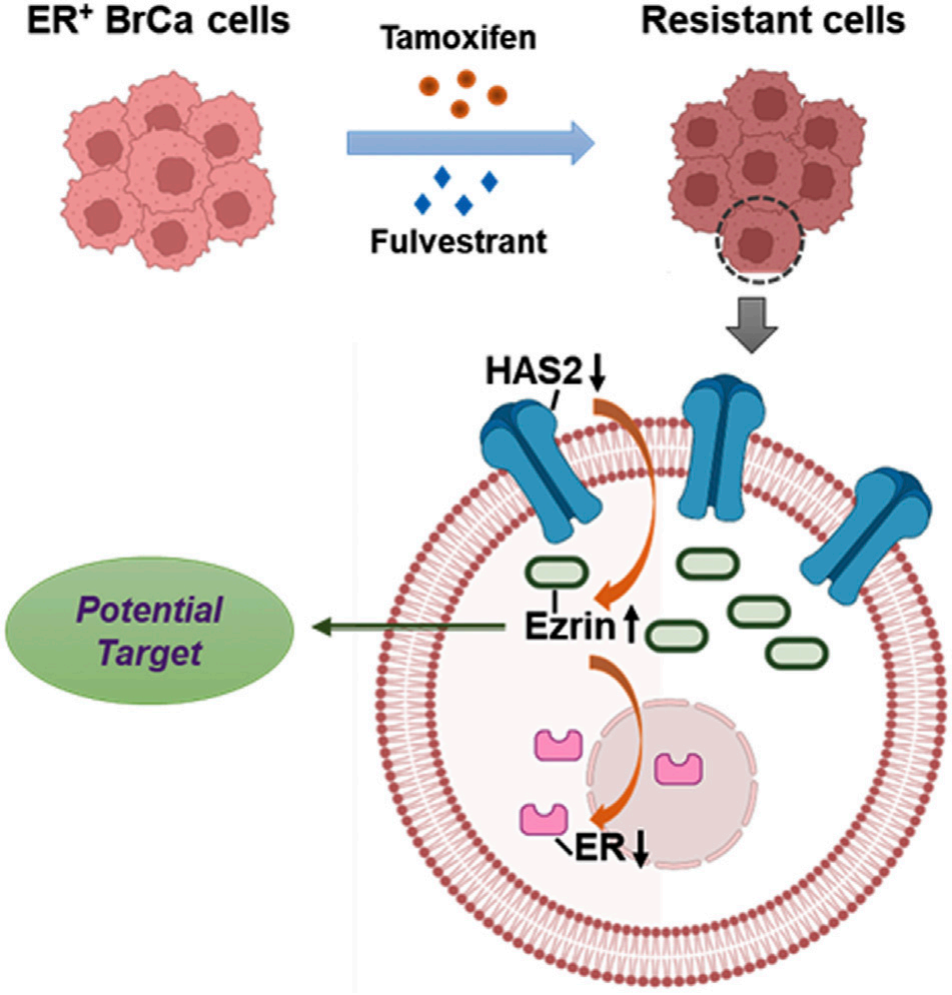
An illustration depicting the role of HAS2-Ezrin-ER axis in endocrine resistance of ER^+^ BrCa.

Endocrine therapy has showed significant advances in antitumor treatments in clinical ER^+^ breast cancer (BrCa) patients. However, the acquired endocrine resistance remains an unsolved problem to medical researches. In this work, we presented a new finding on hyaluronan synthase 2 (HAS2) and the related membrane cytoskeletal cross linker protein, Ezrin, on antiestrogen resistance of receptor-positive (ER^+^) breast cancer cells. We identified the critical significance of HAS2 decrease in the acquired ER^+^ BrCa drug resistance followed by an increase of Ezrin which was in turn responsible for the reduction of ER activity. Our results may indicate a HAS2-Ezrin-ER axis that contributes to the antiestrogens resistance (Figure 6).

Hyaluronate synthases 2 (HAS2) is one of the most important synthases of hyaluronan in vertebrates and is required for embryonic development (Camenisch et al., 2000). It is accepted that HAS2 gene is readily to be regulated by hormone-type effectors (Zhang et al., 2000; Saavalainen et al., 2005; Pasonen-Seppänen et al., 2008) and is related to cell plasticity (Chanmee et al., 2014; Yang et al., 2020). Most importantly, accumulating evidence has demonstrated that HAS2 is overexpressed in various cancers and correlates with a poor prognosis of patients. For instance, the upregulation of HAS2 is associated with tumor progression and metastasis in breast, oral, endometrial cancer, and astrocytoma. Particularly, studies have even demonstrated that HAS2 was involved in regulating antiestrogen sensitivities (Vanneste et al., 2017). However, the mechanism underlying HAS2 and antiestrogen resistance has not been thoroughly investigated until now. In this report, we aimed to identify the role of HAS2 on antiestrogen resistance of ER^+^ BrCa. In contrast to HAS2 or hyaluronan up-regulation in BrCa as reported before (Auvinen et al., 2014), we found a significant decrease of HAS2 expression after endocrine resistance is acquired in estrogen receptor-positive (ER^+^) breast cancer cells, with a predictable down expression of ER. This may point to a different regulation of HAS2 expression in the hormone-positive breast cancer cell lines. In fact, our experiments demonstrated that when HAS2 was restored, the antiestrogen resistance in BrCa cells became sensitive to endocrine treatment, implying that HAS2 could increase the sensitivity of antiestrogen resistant cells. As a membrane-bound protein (Passi et al., 2019), the intracellular segment of HAS2 is recognized to be related to the functions of cytoskeleton proteins (Vigetti and Passi, 2014). We recently reported that the cytoskeletal protein Ezrin existed in the regulatory network of HAS2 mediated resistance to PI3Kα-inhibitory chemotherapy (Yang et al., 2020). We next ask if Ezrin could be involved in the antiestrogen sensitivity regulated by HAS2.

Ezrin is a member of the membrane-cytoskeleton linker Ezrin-Radixin-Moesin (ERM) family of proteins, which are involved in the assembly of specialized domains of the membrane. Studies have showed that ERM proteins were originally characterized as structural components of the cell cortex but were later shown to participate in regulating malignant behaviors of various cancer cells (Clucas and Valderrama, 2014). Additionally, a high level of Ezrin in tumor was reported to be associated with higher metastatic potential and shorter patient survival (Xu et al., 2022). In particular, previous reports found that Ezrin could induce erlotinib resistance of non-small cell lung cancer cells by interacting with epidermal growth factor receptor (Saygideğer-Kont et al., 2016). In this study, we demonstrated that Ezrin was primarily increased in the acquisition of antiestrogen resistance and was negatively correlated with HAS2 expression in BrCa patients. Moreover, our experiments showed that Ezrin was increased after HAS2 was knocked out, while Ezrin was decreased when HAS2 was overexpressed, suggesting that Ezrin could be negatively regulated by HAS2. Actually, previous reports have shown that Ezrin and its binding protein NHERF1 were upregulated in response to estrogen in breast and ovarian cancers (Paradiso et al., 2013), implying that there might be a potential link between Ezrin and estrogen or ER. As the decrease of ER is a hallmark of acquired endocrine resistance that is associated with BrCa recurrence (Nardone et al., 2015; Chi et al., 2019), our study further indicated that Ezrin could negatively regulate ER expression and activation which supports our above hypothesis. Herein, we proposed that a HAS2-Ezrin-ER axis was engaged in endocrine resistance in BrCa. Current understandings agree that ER is reduced upon antiestrogen treatment and our results found that HAS2 was simultaneously decreased. In order to search for an approach that might interfere the HAS2-Ezrin-ER axis for the future potential therapy, we performed inhibiting experiments on Ezrin that was upregulated in the axis. As expected, our results revealed that the knockdown or inhibition of Ezrin effectively restored cell sensitivity to antiestrogens, and subsequently reversed the ERα signaling in endocrine-resistant cancer cells, implying the potential role of Ezrin as a target in overcoming endocrine resistance. However, the *in vivo* role of HAS2-Ezrin-ER axis in mediating anti-estrogen resistance is not yet elucidated, which needs further investigation. Future studies are required to explore the preclinical significance of Ezrin in anti-estrogen resistance.

In summary, we uncovered a novel mechanism for endocrine resistance that includes a HAS2-Ezrin-ER axis governing cellular sensitivity to tamoxifen or fulvestrant in luminal-like ER^+^ BrCa cells (Figure 6). We also found that knockdown or inhibition of Ezrin could sensitize resistant cells to endocrine therapy. These findings may provide a new insight into how the ER-signaling is regulated in response to antiestrogen in which Ezrin might be a potential therapeutic candidate.

## Data Availability

The original contributions presented in the study are included in the article/Supplementary Material, further inquiries can be directed to the corresponding authors.
