# Supplementary material for: HAS2-Ezrin-ER axis plays a role in acquired antiestrogen resistance of ER-positive breast cancer
Source: Front Pharmacol. 2022 Oct 31;13:1031487. doi: 10.3389/fphar.2022.1031487 (PMC9659586; doi:10.3389/fphar.2022.1031487)
Supplement: Supplementary file 1 [file DataSheet2.docx]

**Supplemental Table 1 Sequences of primers**

| **Gene** | **Forward** (5′-3′) | **Reverse** (5′-3′) |
| --- | --- | --- |
| ***HAS2*** | TTATGGGCAGCCAATGTA | ACTTGCTCCAACGGGTCT |
| ***FOS*** | GACTGATACACTCCAAGCGG | CATCAGGGATCTTGCAGGC |
| ***PBX1*** | CAGTGAGGAAGCCAAAGAGG | CAGCTGTTTTGGCAGCATAA |
| ***GAPDH*** | AACGGATTTGGTCGTATTGGG | TCGCTCCTGGAAGATGGTGAT |

**Supplemental Table 2 Clinicopathological features of breast cancer patients in relation to HAS2 and Ezrin expression.**

| Tissue marker | n | HAS2 expression (IOD) | |  | | | | Ezrin expression (IOD) | |  | |
| --- | --- | --- | --- | --- | --- | --- | --- | --- | --- | --- | --- |
|  |  | Median | 95% CI | | P value | Median | | | 95% CI | P value | |
| Age |  |  |  | |  |  | | |  | |  |
| ≤65y | 11 | 241915 | 154413-301681 | | 0.4058 | 132170 | | | 72078-230682 | | 0.2904 |
| >65y | 7 | 236245 | 177706-366619 | |  | 208760 | | | 68921-381081 | |  |
| Sex |  | | | | | | | | | | |
| Male | 1 | 340168 |  | |  | 154423 | | |  | |  |
| Female | 17 | 236245 | 184857-294377 | |  | 134608 | | | 107373-255659 | |  |
| Tumor size, cm |  | | | | | | | | | | |
| ≤2 | 12 | 234755 | 178894-323035 | | 0.7551 | 144515 | | | 97748-300402 | | 0.4307 |
| >2 | 6 | 261436 | 130519-336844 | |  | 148537 | | | 47356-236409 | |  |
| TNM stage |  |  |  | |  |  | | |  | |  |
| I | 2 | 193243 | 171628-214858 | | 0.7019 | 338277 | | | 134608-541945 | | 0.3919 |
| II | 1 | 340168 |  | |  |  | | |  | |  |
| III | 10 | 244222 | 116998-381978 | |  | 154148 | | | 32906-301613 | |  |
| IV | 5 | 241915 | 114065-404267 | |  | 87487 | | | 48360-253809 | |  |
| Lymphnode metastasis | |  | | | | | | | | | |
| Negative | 11 | 255179 | 189622-325732 | | 0.5477 | 154423 | | | 108234-318562 | | 0.2145 |
| Positive | 7 | 236245 | 116157-335047 | |  | 87487 | | | 37530-217561 | |  |
| ER |  |  | | | | | | | | | |
| Negative | 6 | 2707671 | 209568-390286 | | 0.0483* | | 277711 | | 82982-481410 | | 0.0234* |
| Positive | 12 | 192446 | 149565-278125 | |  | 133389 | | | 82283-17555 | |  |

*Indicated statistical signifificance (p <0.05)
