# Supplementary material for: HAS2-Ezrin-ER axis plays a role in acquired antiestrogen resistance of ER-positive breast cancer
Source: Front Pharmacol. 2022 Oct 31;13:1031487. doi: 10.3389/fphar.2022.1031487 (PMC9659586; doi:10.3389/fphar.2022.1031487)
Supplement: Supplementary file 2 [file Image1.PDF]

Supplemental Figures

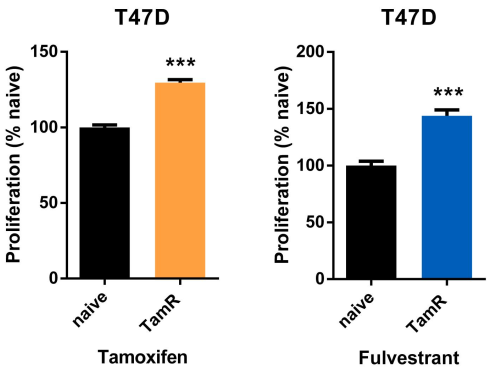

Fig.S1 Characteristics of sensitive and resistant breast cancer cells

Proliferation (3 days) of resistant T47D cells (T47D/TamR and T47D/FulR) was compared with their sensitive counterparts upon tamoxifen (1μM) or fulvestrant (1μM) treatment.

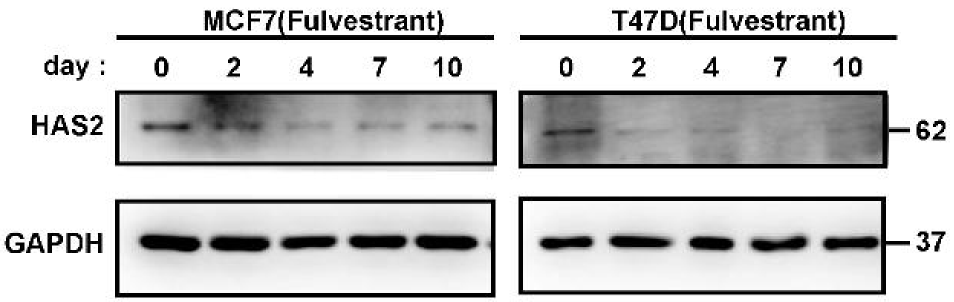

Fig.S2 Changes of HAS2 expression upon antiestrogens treatment

The changes of HAS2 expression in response to fulvestrant were observed by immunoblotting in MCF7 and T47D cells treated for 0, 2, 4, 7, or 10 days.
